# Targeting Protein Kinases and Epigenetic Control as Combinatorial Therapy Options for Advanced Prostate Cancer Treatment

**DOI:** 10.3390/pharmaceutics14030515

**Published:** 2022-02-25

**Authors:** Soghra Bagheri, Mahdie Rahban, Fatemeh Bostanian, Fatemeh Esmaeilzadeh, Arash Bagherabadi, Samaneh Zolghadri, Agata Stanek

**Affiliations:** 1 Medical Biology Research Center, Health Technology Institute, Kermanshah University of Medical Sciences, Kermanshah 6714415185, Iran; sog_bagheri@kums.ac.ir; 2 Institute of Biochemistry and Biophysics, University of Tehran, Tehran 1417614335, Iran; mrohban@ut.ac.ir (M.R.); fatemeh.bostan57@gmail.com (F.B.); 3 Department of Biology, Jahrom Branch, Islamic Azad University, Jahrom 7414785318, Iran; fatima.af1684@gmail.com; 4 Department of Biology, Faculty of Sciences, University of Mohaghegh Ardabili, Ardabil 5619911367, Iran; a.bagherabadi@student.uma.ac.ir; 5 Department of Internal Medicine, Angiology and Physical Medicine, Faculty of Medical Sciences in Zabrze, Medical University of Silesia, Batorego 15 St, 41-902 Bytom, Poland

**Keywords:** tyrosine kinase, serine threonine kinase, epigenetics, signaling pathways

## Abstract

Prostate cancer (PC), the fifth leading cause of cancer-related mortality worldwide, is known as metastatic bone cancer when it spreads to the bone. Although there is still no effective treatment for advanced/metastatic PC, awareness of the molecular events that contribute to PC progression has opened up opportunities and raised hopes for the development of new treatment strategies. Androgen deprivation and androgen-receptor-targeting therapies are two gold standard treatments for metastatic PC. However, acquired resistance to these treatments is a crucial challenge. Due to the role of protein kinases (PKs) in the growth, proliferation, and metastases of prostatic tumors, combinatorial therapy by PK inhibitors may help pave the way for metastatic PC treatment. Additionally, PC is known to have epigenetic involvement. Thus, understanding epigenetic pathways can help adopt another combinatorial treatment strategy. In this study, we reviewed the PKs that promote PC to advanced stages. We also summarized some PK inhibitors that may be used to treat advanced PC and we discussed the importance of epigenetic control in this cancer. We hope the information presented in this article will contribute to finding an effective treatment for the management of advanced PC.

## 1. Introduction

A considerable number of about 2.5% of the human coding genome belongs to the protein kinase (PKs) family, and the mutation and dysregulation of PKs play a critical role in several diseases, including cancers. Due to this, PKs have become one of the leading pharmacological drug targets in the 21st Century [1], and protein kinase inhibitors (PKIs) are a promising new class of therapeutic agents [2]. The availability of the potent inhibitors of understudied kinases could greatly aid the discovery of uncovering new targets for drug development [3]. Ferguson et al. provided an overview of the novel targets, biological processes, and disease areas that kinase-targeting small molecules are being developed against and evaluated the strategies and technologies that generate highly optimized kinase inhibitors [4]. Recently, Klaeger et al. performed a comprehensive analysis of 243 kinase inhibitors that are either approved for use or in clinical trials [5,6]. According to the latest update (21 January 2022) of the PK Inhibitor Database (PKIDB) [7], 72 FDA-approved medicinal products target different types of PKs. Eight of these PKIs were approved in 2021 and one in 2022. The PKIDB shows that although most of these drugs are indicated as various cancer therapeutics (solid and nonsolid tumors); however, information on the use of these drugs in prostate cancer (PC) is still insufficient. 

PC is an endocrine-related disease [8], ranked as the most commonly diagnosed malignancy [9]. The male hormone androgens play a crucial role in PC progression through androgen receptor (AR) activation [10]. This issue has made AR an important therapeutic target for PC therapy [11]. The first-line treatments for metastatic PC are androgen-deprivation therapy (ADT) and AR-targeting therapy, but secondary resistance coupled with enhanced metastatic potential is a crucial challenge in these treatments [12]. Mounting evidence suggests that PKs play a crucial role in tumor growth, proliferation, and metastasis in PC. Furthermore, they may be responsible for resistance to standard treatments [13,14,15]. Additionally, recent discoveries indicated the complex crosstalk between the PKs and epigenetic events and critical biological pathways, including AR signaling pathways [12,16]. Therefore, a deep understanding of how genetic and epigenetic mechanisms regulate the progression of PC appears to be essential to design therapeutic agents for PC patients [16]. For these reasons, hopeful studies are now underway with epigenetic modulators [17] and kinase inhibitors [13,18] as combination therapy options to gain selectivity and overcome resistance. In this review article, we focused on the role of PKs and epigenetic processes in PC progression and discussed the new advancements for the management and treatment of this cancer by controlling epigenetics and targeting the PK family. 

## 2. A Brief Introduction to the Family of PKs 

PKs catalyze phosphorylation reactions to regulate the enzyme activity, protein functioning, and signal transduction pathways by transferring a phosphate group to an acceptor amino acid of the substrate protein [19]. They play a crucial role in cellular processes, such as metabolism, motility, and cell division [20,21,22]. The dysregulation of PK activity is associated with the pathogenesis of several diseases, including cardiovascular [23,24], autoimmune, and inflammatory [25] diseases, as well as cancers [26]. The first classification of PKs is related to the efforts of Tony Hunter and Steven Hanks [27,28]. This classification was extended by Manning et al. [21]. Based on the phosphate acceptor amino acid specificity, PKs can mainly be divided into two subdivisions: protein–serine/threonine kinases (Ser/Thr kinases) and protein–tyrosine kinases (Tyr kinases). Additionally, comprehensive sequence analysis of PKs led to a classification system consisting of nine main groups in phylogenetic trees: calcium/calmodulin-dependent PK (CAMK), AGC (containing PKA, PKG, and PKC families), Tyr kinase (TK), TK-like kinase (TKL), CMGC (containing cyclin-dependent kinase (CDK), mitogen-activated PK (MAPK), glycogen synthase kinase-3 (GSK3), and CDC2-Like PK (CLK) families), casein kinase 1 (CK1), STE (homologs of yeast Sterile 7, Sterile 11, and Sterile 20 kinases), receptor guanylate cyclases (RGC) group, and atypical PKs [21].

Currently, among the 497 typical kinase domains in the human genome, 284 kinase structures have been determined experimentally, either as apo or in complexes with inhibitors or ATP. Generally, the protein kinase fold consists of two domains: an N-terminal domain and a C-terminal domain. The N-terminal region consists of an alpha helix called C-helix, as well as five beta-sheet strands, and the C-terminal domain commonly contains five or six helices, namely the D, E, F, G, H, and I alpha helices. A deep cleft created by the N-terminal and C-terminal lobes in the middle region of the protein forms the active site for ATP-binding. In addition, the activation loop is one of the most important regions that helps ATP and the substrate bind in the enzyme’s active site. The activation loop includes the Asp–Phe–Gly motif called the “DFG motif,” which adopts an extended unique orientation in the active state conformation of the enzyme and several types of folded conformations in an inactive state [29]. 

**Figure 1:**
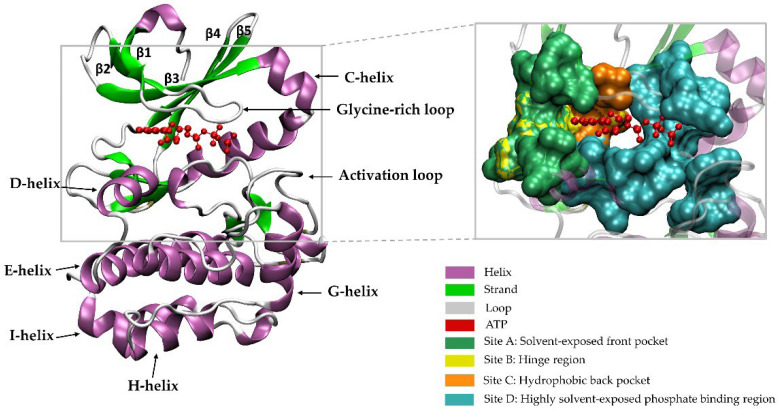
The crystal structure of the Aurora A complex with ATP (PDB:5DNR). The protein’s N-terminus is composed of C-helix, β1 to β5 strands, and a glycine-rich loop, while the C-terminus is formed by helices D, E, F, G, H, and I, the catalytic loop, and the activation loop. Figure produced with visual molecular dynamics (VMD) software [32].

Figure 1 shows the structure of a PK, namely, Aurora A kinase (AURKA) (PDB: 5DNR), and four key sites A–D on the surface binding groove (Figure 1). Site A is the solvent-exposed front pocket (composed of residues 137, 139, 157, 212–216, 220, 224, and 264–266) and site B is the hinge region (residues 210–216) that mainly focus on the hydrogen bonding network. Site C is the hydrophobic back pocket, which is not conserved and identified as the selectivity pocket, and is present in most of the kinases, created by residues 210, 211, 147, 160, and 194 in AURKA. Site D is a highly solvent-exposed phosphate-binding region (formed by amino acid residues 143, 144, 162, 164, 178, 181, 194, 208, 255, 258, 260, 261, 263, 271–275, and 277). Site D is relatively larger compared to site A [30]. Most drugs that bind to the ATP site are considerably hydrophobic and inhibit kinase catalytic activity [31]. However, structural analysis suggested that the solvent-exposed sites A and D, located outside the ATP binding site, could be used to improve the pharmacokinetic properties of lead compounds [30].

Recently, Modi and Dunbrack presented a web resource called Kincore (the Kinase Conformation Resource) that automatically organizes a collection of all PK structures and assigns conformational state and inhibitor type tags. They identified eight active and inactive functional states of the DFG motif. Additionally, they classified the inhibitor type bound to each kinase domain into five categories: type 1, 1.5, 2, 3, and allosteric. Type 1, 1.5, 2, and 3 inhibitors bind to the ATP site, the ATP binding site + a portion of the C-helix region, the ATP binding site + the C-helix region, and the C-helix region, respectively. Allosteric inhibitors bind elsewhere. By combining the classification of the DFG motif conformation and inhibitor types, over 200 inhibitors were found that bind to multiple states of kinases [29].

### Catalytic and Non-Catalytic Activities of PKs

Despite the success of targeted kinase inhibitors in responsive patients, their tumors almost show resistance over time, leading to disease progression and a central challenge for clinical care [33]. Thus, multiple strategies are required to overcome this resistance. A critical approach is inducing and stabilizing inactive kinase forms allosterically [34]. Although PKs are known primarily for their ability to phosphorylate protein substrates, accumulated evidence has recently suggested that most human kinases have non-catalytic activity beyond catalysis through their scaffolds. The non-catalytic activity of PKs involves the allosteric regulation of other kinases or enzymes through protein–protein interactions, assembly of signaling complexes, or even transcriptional regulation via direct binding to DNA or interaction with a transcriptional factor [35]. These non-catalytic activities play a critical role in normal cellular activities and diseases, especially in mediating drug resistance to kinase inhibitors [35]. Most FDA-approved PK inhibitors inhibit kinase catalytic activity upon binding to the ATP binding site. Recently, accumulated evidence has suggested that small molecules modulating the non-catalytic functions of kinases can emerge as new promising therapeutic strategies for various diseases. To date, classes of agents have emerged that can regulate the non-catalytic function of kinases. Orthosteric and allosteric kinase inhibitors, protein degraders, and protein−protein interaction blockers are three categories of these modulators [31]. 

## 3. PK Targeting Tools

Irregular signaling pathways are a hallmark of cancer [36]. Therefore, it is not surprising that PKs are the most drug targets, after the G-protein-coupled receptors [35]. Currently, several tools are available for targeting PKs, each with their advantages and disadvantages. One of these methods is the use of small-molecule kinase inhibitors, which has been widely studied and has been successful in the treatment of various cancers [35]. Accordingly, the FDA has approved 70 small-molecule kinase inhibitors for application in oncology [37]. However, despite their advantages, such as the ability to target multiple cell survival pathways, ease of oral administration, and low production costs, the clinical use of these inhibitors faces a variety of challenges, including cytotoxicity, chemotherapy resistance, and off-target effects [35]. Another method that has been studied to inhibit kinases is the use of synthetic peptides that, despite their advantages, such as high specificity, they have some drawbacks, such as poor pharmacokinetic and biodistribution parameters [38,39]. Short interfering RNA (siRNA), also referred to as RNA interference (RNAi), is a well-known technology that has shown promising therapeutic results in cancer treatment [40,41]. Numerous studies showed that the concomitant use of siRNA and TK inhibitors (TKIs) could sensitize resistant cells to chemotherapy [42,43]. In addition, other evidence revealed that the use of siRNA against various kinases had anticancer effects and significantly reduced the chemotherapy resistance in different cancer cells. Some related studies in this field include studies on polo-like kinase (PLK) [41,44], focal adhesion kinase (FAK) [45], PKB/Akt [46], B-RAF [47], receptor tyrosine kinase-like orphan receptor-1 (ROR1) [48], AURKA [49], eukaryotic elongation factor 2 kinase (EF2K) [50], pyruvate kinase M2 (PKM2) [51], and CDK8 [52]. Nevertheless, despite its special advantages, such as its high degree of specificity [53,54], siRNA faces numerous challenges, such as systemic toxicity, obstacles with delivery to various tissues, and a high degradation rate in the presence of serum proteins and enzymes [40,42,43]. Another way to inhibit kinases is the use of kinase-targeted antibodies, which has been reported to be effective in various cancers [34], for instance, monoclonal antibody against epidermal growth factor receptor (EGFR) in colorectal cancer [55] and against HER2 in breast cancer [56,57]. The inhibition of kinases with monoclonal antibodies, despite having advantages, such as high specificity, presents adverse effects, such as allergic reactions and the development of various cytotoxicities [58]. Another tool for blocking kinases is the use of proteolysis-targeting chimera (PROTAC) technology. PROTACs bind to proteins of interest and use E3 ligase to degrade the entire target protein via the ubiquitin–proteasome pathway [59]. However, as with other methods, PROTAC technology faces challenges, such as acquired and intrinsic resistance to drugs in cancer cells [60]. The use of natural products and probiotics are other tools that have been considered as potential kinase inhibitors in recent years. Various natural products, including curcumin [61], green tea extracts [62], luteolin [63], quercetin [64,65], and resveratrol [66], have shown inhibitory activity against different kinases in various cancers, and several mechanisms have been proposed for the effect of these compounds on the reduction of kinase mutations. The use of natural products also has its own problems due to issues such as accessibility, sustainable supply, and intellectual property constraints [67]. Various probiotics and their metabolites have also been studied as kinase inhibitors, and research in this field is expanding [68,69].

## 4. PKs and PC Progression

In general, studies on kinases have suggested both anti-cancer and pro-cancer roles for them, and this dual role has been attributed to having different subunits, the localization of isozymes in different cell subunits, and the different contexts of their activity. In this article, the complexity of the role of kinases is neglected, and the focus is on reports that have shown the pro-cancer role of them. In addition, although the role of many kinases in PC progression has been reported, such as EGFR [70], EphA2 [71,72], Janus kinase 1 (JAK1) [73], JAK2 [74], c-Jun N-terminal kinase (JNK) [15], MAPK4 [75], protein tyrosine kinase 6 (PTK6) [76], ribosomal S6 kinases (RSKs) [77], vascular endothelial growth factor receptor 3 (VEGFR-3) [78], etc., only a few of them have been cited as examples, and the mechanism of their effect on PC progression is discussed in detail.

### 4.1. AMP-Activated PK (AMPK)

AMPK belongs to Ser/Thr PKs, which is activated by enhanced intracellular AMP concentrations [79]. AMPK is a main cellular energetic biosensor that regulates a large number of metabolic pathways activated by nutrient (glucose) deprivation, low oxygen gradients, mitochondrial dysfunction, oxidative stress, and cytokines [80,81]. Activated AMPK promotes energy-sparing mechanisms and induces anti-apoptotic functions. As a result, it allows cells to survive for a very long time in very hostile conditions [81]. Multiple studies showed a positive correlation of AMPK phosphorylation/activation with the Gleason score and disease progression in PC patients [82,83,84]. AMPK activity is regulated by androgen and upstream kinases, including the CAMK kinase 2 (CAMKK2) in PC [85,86]. Indeed, androgen enhances AMPK activation and autophagy, thereby promoting PC growth [87]. New findings on the association between AMPK and metabolic reprogramming showed that the AMPK/GSK3β/β-catenin cascade might upregulate the cell-migration-inducing protein (CEMIP), which could drive the migration and invasion of anoikis-resistant PC cells via increasing pyruvate dehydrogenase kinase isoform4 (PDK4)-associated metabolic reprogramming [88]. 

Macropinocytosis is a process of non-selective swallowing of extracellular material through the ruffling of the plasma membrane [89]. Ras-related C3 botulinum toxin substrate 1 (RAC1-GTP) and phosphatidylinositol (3,4,5)-trisphosphate (PIP3) are necessary for macropinosome formation [90,91]. In fact, by macropinocytosis, cancer cells with activating mutations in RAS use extracellular proteins as a fuel when amino acids are limiting [92]. PIP3 is produced by phosphoinositide 3-kinases (PI3K) and converted to (phosphatidylinositol (4,5)-bisphosphate (PIP2) by phosphatase and the tensin homolog (PTEN) [93]. PTEN is a tumor suppressor gene that is most frequently disrupted in PC [94,95] and is correlated with an increased risk of metastasis and resistance to castration [94,96]. Indeed, macropinocytosis is a cancer-related phenotype caused by a loss of PTEN function. As a regulator of PI3K signaling, the loss of PTEN leads to Akt overactivity, followed by reduced apoptosis, uncontrolled cell proliferation, and increased tumor angiogenesis [97]. Recent studies have revealed that the loss of PTEN is not sufficient to induce macropinocytosis in PC cells and that AMPK activation is essential. In fact, AMPK activates RAC1, which is essential for membrane ruffling in macropinocytosis [98].

### 4.2. Protein Kinase A (PKA)

PKA, also known as cAMP-dependent PK, is a member of Ser/Thr PKs that regulates the signal transduction of G-protein-coupled receptors through its binding to cAMP [99], and its role in the onset and progression of many tumors has been demonstrated [99,100]. cAMP is a second messenger that is involved in various cellular functions, including the ion channel activation, gene expression, cell growth and differentiation, and apoptosis [100]. 

Progress toward castration resistance is a crucial problem in the treatment of advanced PC. Numerous evidence suggests that PC cells develop castration resistance by activating multiple molecular pathways, including AR and PKA [101]. In the absence of androgen, increasing levels of cAMP/PKA pathways have been shown to increase the expression of AR and prostate-specific antigen (PSA) proteins in PC cells [102], which, in turn, can lead to increased androgen signaling, resulting in cell proliferation and subsequent castration-resistant PC (CRPC) [103]. The type II beta regulatory subunit of PKA, cAMP-dependent protein kinase type II–beta regulatory subunit (PRKAR2B), is highly expressed in CRPC and is involved in tumor proliferation and metastasis. A new study has revealed that PRKAR2B enhanced the expression level of hypoxia-inducible factor 1α (HIF-1α), a crucial moderator of the Warburg effect, thereby promoting tumor growth [104].

Recent studies have shown the phosphorylation of different proteins by PKA that suppresses apoptosis or stimulates invasion and metastasis. PKA activation is essential for the phosphorylation of heat-shock protein 90 (Hsp90), which binds to the ligand-free AR in the cytoplasm and restricts its entry into the nucleus. However, new findings suggest that the PKA-mediated phosphorylation of Thr89 residue of Hsp90 can lead to the release of AR from Hsp90, subsequently binding AR to Hsp27 and its migration to the nucleus [103]. Some studies have demonstrated that PKA phosphorylates caspase-9 induces the disassembly of the large and small subunits of caspase-9 and prevents its self-processing, thus inactivating caspase-9 and suppressing the progression of apoptosis [105]. Another study indicated that the calcitonin receptor, which enhances PC cell invasion, activated PKA that phosphorylated the tight junction proteins ZO-1 and claudin 3, destabilizing the tight junctions and increased PC cell invasion [106]. Other studies revealed other aspects of the association between PKA and PC progression, including the association between PKA and angiogenesis [107], the reduction of the Ca^2+^-store content in the endoplasmic reticulum (ER) [108], and the mediating of the tumor-associated macrophage polarization phenotype [109].

### 4.3. Protein Kinase B (PKB)

PKB, also known as Akt, is a member of the Ser/Thr kinases whose protein expression and activity have been shown to increase in many tumors and tumor cells [110,111]. Several kinases, including 3-Phosphoinositide-dependent kinase 1(PDK1) and mammalian target of rapamycin (mTOR) complex 2 (mTORC2), activate Akt. An increase in the PIP3 levels by PI3K causes the uptake of Akt into the plasma membrane and its activation [112]. The association of Akt activation with the PC progression from an androgen-dependent stage to an androgen-independent stage has been shown [113,114]. Akt enhances the androgen-independent survival of prostate tumor cells by regulating AR expression and activation [113]. Akt phosphorylates the residues Ser213 and Ser791 of AR, leading to AR signaling and cell survival [115]. The role of Akt in the PI3K/Akt/mTOR pathway has been demonstrated in many studies, so this pathway has been proposed as the main regulatory factor of pro-survival/anti-apoptotic pathways in the absence of AR signaling [116]. Studies on different human PC cell lines have shown that the inhibition of PI3K or expression of the dominant negative mutant of Akt inhibits invasion and decreases the expression of urokinase-type plasminogen activator (uPA) and matrix metalloproteinase-9 (MMP-9), which are markers of cell invasion [117]. 

Various studies have revealed different aspects of the PKB’s effect on cancer progression. For instance, the overexpression of fatty acid synthase (FAS) in PC tissues is associated with Akt phosphorylation and nuclear accumulation. FAS is an essential metabolic enzyme associated with the synthesis of membrane phospholipids in cancer cells, high levels of which are expressed in human epithelial cancers, especially those with poor prognosis [118]. The forkhead box transcription factor FoxO3a is known to be a tumor suppressor whose activity has been shown to be negatively regulated by Akt through post-translational modifications [119,120]. Indeed, phosphorylation at Ser253 increases the accumulation of FoxO3a and its binding chaperone protein 14-3-3 in the cytosol, thus reducing its level in the nucleus [119]. Subsequent studies disclosed that Par-4 is one of the crucial FoxO3a transcriptional targets, and par-4 activation is necessary to induce apoptosis in CRPC cells [120]. Furthermore, other reports indicated that the inhibition of FoxO3a accelerated PC progression in the transgenic adenocarcinoma of mouse prostate (TRAMP) mice, which was correlated with increased proliferation and survival markers [121]. 

### 4.4. Protein Kinase C (PKC)

PKC belongs to Ser/Thr PKs, in which its different isoforms play significant roles in the cell cycle and cell death, and changes in their expression or activity have been identified in human diseases [122,123]. The effect of PKC on the cell cycle is highly context-dependent and varies depending on the specific isoenzyme involved and other factors, such as the time and duration of enzyme activation [122]. Some studies suggested that PKC-α, PKC-ε [124], and the atypical PKCs (aPKCs), PKC-λ/ι [125] and PKC- ζ [124,126], preferably induce cell proliferation and survival, while PKC-δ regulates apoptosis [127].

It has been shown that AR phosphorylation at the Ser-578 residue, which is attributed to PKC [128], may cause PC progression [129]. Immunohistochemical studies of human PC tissue microarrays showed that the PKCε expression levels were associated with PC aggressiveness. Further studies on human PC, human PC cell lines, and PC developed in TRAMP mice illustrated those signal transducers and activators of transcription 3 (STAT3) that are primarily active in a wide range of human cancers, including PC, which interact with PKCε and is phosphorylated at Ser727. The inhibition of PKCε expression inhibited STAT3Ser727 phosphorylation, followed by decreased DNA binding and STAT3 transcriptional activity, as well as reduced cell invasion. These results suggest that PKCε activation is necessary for STAT3 activation and PC progression [130]. 

PKCε collaboration with PTEN loss for PC development has been demonstrated in a mouse model. The overexpression of PKCε and PTEN loss, individually and synergistically, positively regulates chemokine (C-X-C motif) ligand 13 (CXCL13) production. In addition, the disruption of CXCL13 or its receptor in PC cells affects its tumorigenic and migratory properties. The role of the chemokine CXCL13 and its receptor, C-X-C chemokine receptor type 5 (CXCR5), has been reported to be a major factor in the progression of many cancers, including PC [131]. Various studies have described various mechanisms by which PKCε is involved in the progression and metastasis of PC, including enhancing aerobic glycolysis [132], the activation of nuclear factor kappa-light-chain-enhancer of activated B cells (NF-κB)) [131], phosphorylation of Vimentin [133], and interaction with BCL2 associated x, apoptosis regulator (Bax) [134].

Available evidence suggests that both aPKCs, as with other PKC isoforms, play pleiotropic context-dependent roles, and some studies have reported pro-tumorigenic roles for them [124]. Vimentin overexpression is known as a hallmark of the epithelial–mesenchymal transition (EMT), and the molecular dynamics of Vimentin intermediate filaments (VIFs) play a significant role in metastasis [135]. New findings show that aPKCs activate Vimentin by phosphorylating Ser33, Ser39, and Ser56 residues in Vimentin, resulting in VIF disassembly, which contributes to PC cell metastasis [136]. In addition, both PKC-ι and PKC-ζ have been shown to induce cell survival through the NF-κB/PI3K/Akt pathways [125].

### 4.5. Protein Kinase D (PKD)

PKD is a family of Ser/Thr kinases belonging to the CAMK superfamily. The physiological functions and regulatory mechanisms of PKD, including the regulation of gene expression, protein/membrane trafficking, cell proliferation, survival, migration, and angiogenesis, are well documented [137]. Three PKD isoforms, PKD1, PKD2, and PKD3, are stimulated by various extracellular stimuli and transduce cellular signals that affect many aspects of primary cellular function [138]. Dependent on subcellular localization, PKD isoforms control various processes, including cell signaling, Golgi transport, and the oxidative stress response [139]. Further studies at the cellular level and in animal models have shown the vital role of PKD in numerous pathological conditions, including cancer [138]. An in-depth in vitro migration study on Panc1 pancreatic cancer cells to clarify the role of PKD in cancer cell migration indicated that the absence of each PKD isoform exerts a considerable effect on cell speed and migration persistence, and that the absence of PKD1 is associated with a significant increase in Panc1 cell deformability [140]. 

Current findings suggest a potential tumor-promoting function for selective PKD isoforms in PC [141]. It has been shown that PKD3 interacts with sterol regulatory element-binding protein 1 (SREBP-1) and consequently promotes cell proliferation via lipid metabolism in PC cells [142]. Some evidence has shown that PKD2 and PKD3 enhance NF-κB signaling and uPA expression/activation, that are critical for PC invasion [143]. EMT and cell migration play a key role in the onset and progression of diverse malignancies, including PC. Different PKD isoforms act differently in these processes, with PKD1 inhibiting EMT and cell migration, but PKD2 and PKD3 induce these processes [144]. Previous studies have indicated that a PKC/PKD pathway protects PC cells against phorbol ester-induced apoptosis via elevating the extracellular signal-related kinase1/2 (ERK1/2) and NF-κB transcriptional activities [105]. Furthermore, a more recent report revealed that PKC and PKD play a significant role in PC cell migration induced by CXCL12 chemokine [145].

### 4.6. DNA-Dependent Protein Kinase (DNA-PK)

DNA-PK is a nuclear Ser/Thr PK [146], which plays a significant role in the repair of double-strand breaks [147]. In addition to interfering with DNA repair, DNA-PK plays a regulatory role in transcription by phosphorylating transcription factors, thereby regulating their functions [148]. Recent studies have shown new, different roles beyond DNA repair for the DNA-PK catalytic subunit (DNA-PKcs) in cancer, including its involvement in cell cycle progression, metastasis, treatment resistance, metabolic dysregulation, and immune escape [147].

DNA-PK is well documented to control tumor metastasis and progression in PC by various mechanisms, such as interaction with AR or the phosphorylation of insulin-like growth factor (IGF)-binding protein 3 [149]. A recent study was performed to recognize the kinases that drive PC progression, and tumor samples were collected from 545 patients with high-risk diseases. The results of this study identified DNA-PK as the most important kinase related to metastatic progression in high-risk PC. It also showed that DNA-PK mainly drives PC by regulating the transcription of Wnt signaling members [150]. Some studies revealed that DNA-PK and mTOR, through localization in chromatin at specific regulatory sites, function as AR cofactors in PC cells. In fact, the nuclear localization of mTOR and DNA-PK expression, both of which increase in advanced PC, are associated with metastasis and reduced overall survival [151,152].

### 4.7. CDC2-Like Protein Kinase (CLKs)

The CLK family, known as signaling kinases [153], consists of four isoforms, including CLK1, CLK2, CLK3, and CLK4 [154], with specific and conserved ATP binding sites like other kinases. CLKs can phosphorylate serine, threonine, and tyrosine residues. Therefore, dual-specificity kinase activity is observed in the CLK family [155]. The structure of CLKs consists of domains N and C, which are connected by the hinge region, β-strands, and α-helices distributed between the N and C regions as a catalytic domain [156]. CLKs and then bind to pre-mRNA and stabilize the serin and arginine-rich splicing factors 1–12 (SRSF1–12) are phosphorylated by the interaction of spliceosome components and spliceosome assembly [157]. The expression levels of CLK isoforms are different between cell types and problems, such as prostate, testes, brain, leukocytes, muscle, liver, lung, kidney, and thyroid [158]. 

In PC, the expression level of prostate-associated gene 4 (PAGE4) increases. PAGE4 is an intrinsically disordered protein (IDP) with significant roles in the development and differentiation of PC. PAGE4 is not detectable in the normal adult gland [159]. Hence, it has the hallmarks of a proto-oncogene. Homeodomain-Interacting PK 1 (HIPK1) is an element of the cellular stress-response pathway and can phosphorylate the Ser9 and Thr51 of PAGE4, but phosphorylation in Thr51 is critical. In addition, hyperphosphorylation occurs at multiple Ser/Thr residues by CLK2. PAGE4 phosphorylation by HIPK1 increases c-Jun activity (a component of the stress–response pathway), whereas phosphorylation by CLK2 decreases this activity. Therefore, these two kinases have opposite functions [160]. Androgen receptor, a crucial therapeutic target in PC, is negatively regulated by activator protein-1 (AP-1). The formation of transcription factor Ap-1 is related to the heterodimerization of the proto-oncogene c-Jun with c-Fos [161]. Furthermore, CLK2-PAGE4 shows a low affinity for transcription factor Ap-1. Therefore, the conformational dynamics of PAGE4, which are induced by phosphorylation, may play a role in modulating alterations between PC cell phenotypes [159,160].

### 4.8. Serine-Argnine Protein Kinase 1 (SRPK1)

Ser-Arg PKs (SRPKs) can phosphorylate serine residues located in the rich region of Arg/Ser or Ser/Arg dipeptides motifs. Therefore, the SRPK family can regulate alternative splicing as SR splicing factor phosphorylation [162]. SRPK1 phosphorylates SR proteins, such as SRSF1 (splicing factor 1), and regulates RNA maturation, protein phosphorylation, cell cycle progression, the regulation of viral genome replication, chromatin reorganization, and immune response. Therefore, various functions of SRPK1 in cellular processes distinguish it from other kinases [163,164]. SRPK1, as with other Tyr kinases, consists of two conserved kinase domains. The large lobe of the C-terminal domain is the substrate-binding site and consists of α-helices, whereas the small lobe of the N-terminal domain comprises β-strands and is an ATP binding site [165].

Vascular endothelial growth factor-A (VEGF-A) induces angiogenesis, which is required for tumor growth. The level of VEGF increases in the urine and plasma of advanced stages of PC. The two families of VEGF isoforms of pro-angiogenic and anti-angiogenic are produced during the alternative splicing of VEGF-A pre-mRNA with the dominant isoform of VEGF_165_b. VEGF_165_b is anti-angiogenic. In PC, only the pro-angiogenic isoform is upregulated. SRSF1 phosphorylation by SRPK1 can control VEGF splice isoforms. The overexpression of SRPK1 is observed in PC progression [166,167,168]. Therefore, the inhibition of SRPK1 can switch to the expression of the anti-angiogenic isoform [164,166]. 

### 4.9. Pyruvate Kinase M2 (PKM2)

PKM2, a key glycolysis enzyme, is overexpressed in many tumor cells and plays a critical role as a regulator in tumor metabolism [169]. PKM2 has been demonstrated to be overexpressed in PC and promotes PC metastasis via ERK-cyclooxygenase (COX-2) [170]. Other studies have shown that there is a significant positive correlation between PKM2 nuclear localization and PC aggressiveness; also, the pharmacological targeting of PKM2 nuclear translocation disrupts the metastatic dissemination of PC cells in SCID mice [171]. In addition, the comparison of serum-derived exosomes from PC patients with healthy men showed that increased exosome PKM2 expression was associated with metastasis. A recent study identified the exosome-mediated transfer of PKM2 from PC cells to bone marrow stromal cells as a new mechanism by which exosomes derived from the primary tumor promote the formation of pre-metastatic niches [172].

### 4.10. T lAK Cell Originated PK (TOPK)

T lAK Cell-Originated PK (TOPK) plays a role in the mitotic progression and regulation of the cell cycle, and is expressed in both the nucleus and the cytoplasm. Due to its high homology to mitogen-activated protein kinase kinase 3 (MKK3), TOPK is a MAPK kinase (MAPKK) and is a dual-specificity Ser/Thr kinase [173]. It seems that TOPK plays a role in the activation of Akt, ERK, and JNK due to the dual-specificity family of kinases. Akt is activated when PTEN is phosphorylated and deactivated by TOPK [174]. The tissues with high levels of proliferation overexpress TOPK, while the expression of TOPK is minimal in differentiated cells. Therefore, the invasion, aggressiveness, and metastatic growth of tumors are linked to the overexpression of TOPK [175]. CDK1/cyclin B1 complex phosphorylates the TOPK in Thr9; hence, TOPK is functionally activated and can destabilize the tumor suppressor P53 and damage mitosis. Indeed, the overexpression of TOPK leads to aberrant entry into the mitotic phase by phosphorylating histone H3 at Ser10 via bypass of the G2/M checkpoint, downregulation of p53 (tumor suppressor), and upregulation of the CDK inhibitor p21 [174]. Conversely, the inhibition of TOPK activity leads to a reduction in the phosphorylation and activation of MAPK, reducing the inhibition of Akt activation and inhibiting the expression of mutant p53; therefore, the tumorigenic properties are impaired. Recently, Alhawas et al. reported the direct role of TOPK in the regulation of an alternatively spliced AR variant, ARv7, and the driving of androgen-independence in PC cells [176].

### 4.11. Src Family Kinases (SFKs)

**Figure 2:**
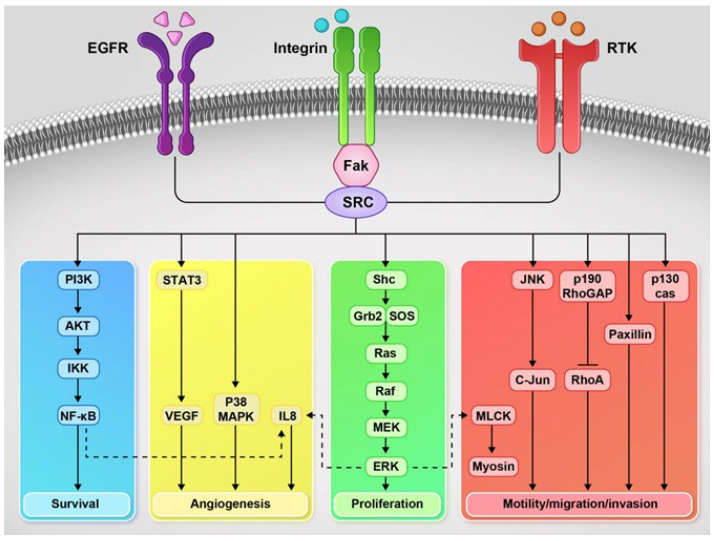
Src signaling. FAK, focal adhesion kinase; RTK, receptor Tyr kinase; PI3K, phosphatidylinositol 3-kinase; Akt, PKB; IKK, IkappaB kinase; NF-κB, nuclear factor kappa light chain enhancer of activated B cells; STAT3, signal transducer and activator of transcription 3; VEGF, vascular endothelial growth factor; MAPK, mitogen-activated PK; IL-8, interleukin 8; Shc, Src homology 2 domain-containing; Grb2, Growth factor receptor-bound protein 2; SOS, son of sevenless; MEK, mitogen-activated protein kinase kinase; ERK, extracellular signal-regulated kinase; MLCK, myosin light chain kinase; JNK, c-Jun N-terminal kinase; RhoGAP, Rho GTPase-activating protein; and CAS, Crk-associated substrate).

Src family kinases (SFKs), the largest family of non-receptor Tyr kinases, are responsible for signal transduction during cell differentiation, adhesion, and migration during normal cellular processes. Due to these roles, SFK-activated signaling pathways are involved in angiogenesis, motility, invasion, and tumor adhesion (Figure 2). Recent evidence suggests that Src activity may play a prominent role in cancer progression, including PC. Drake et al. demonstrated a significant upregulation of Tyr phosphorylation in CRPC. Additionally, they found that the increased expression of Src and AR can synergistically drive the frank of prostate carcinoma [177].

### 4.12. Focal Adhesion Kinase (FAK)

FAK, a member of the non-receptor Tyr kinase located at the extracellular matrix cell adhesion site, is associated with the development and progression of cancer. FAK regulates downstream signaling pathways on the cell-extracellular matrix of integrins, growth factor receptors, cytokine receptors, and G-protein-coupled receptors. It has been found that the development of tumor malignancy is often associated with disturbance in these signaling cascades [178]. FAK, an essential mediator of integrin-associated signaling, is a well-established example of the non-catalytic function of PKs. Following integrin clustering, FAK acts as a scaffolding protein to assemble focal adhesion by interacting with the integrin-binding [31]. Studies by Marcellus et al. showed that the activation of FAK in the metastatic PC3 cell line is an essential factor for the colony formation in PC3 cells, thus affecting cell motility [179]. Additionally, Slak et al. investigated the role of FAK in cell migration and demonstrated that the metastatic potential of PC correlates with its intrinsic migratory capacity, and the metastatic potential correlates with the FAK expression and activation. Moreover, they reported that the autophosphorylation of FAK is adhesion-dependent in PC3, whereas Tyr861, as the second site of phosphorylation, an Src-specific site, is uncoupled from adhesion-dependent events. Significant inhibition of prostate cell migration is achieved by inhibiting the FAK/Src signaling pathway (Figure 2), demonstrating that cell migration depends on signals emanating from this pathway [180]. In a study on 100 patients with prostate adenocarcinoma, a strong functional interaction between FAK and MMP-9 has been shown and, consequently, enhanced the angiogenesis, invasion, and progression of prostate adenocarcinoma [181]. Taken together, these studies suggest that, for patients with prostate adenocarcinomas, FAK/Src may be considered as new therapeutic targets. Further investigations are needed to clarify their importance.

### 4.13. Cyclin G-Associated Kinase (GAK)

Cyclin G-associated kinase (GAK), also known as auxilin II, is a Ser/Thr kinase, which is homologous to auxilin I, except that there is a kinase domain at the N terminus of GAK [182,183]. GAK, which is localized in the cytoplasm (particularly at the trans-Golgi network) and nucleus [184], plays a significant role in membrane trafficking and the sorting of proteins [185,186]. GAK is localized principally in the nucleus in cancer cells and nuclear GAK overexpression was reported in surgical specimens from PC patients [187]. GAK overexpression was identified in over 90% of androgen independent (AI) tumor biopsies from PC patients [188]; a positive correlation between GAK expression and the Gleason score in surgical specimens from PC patients was reported [186]. GAK has been shown to be involved in the progression of cancer to AI [188], although this is not because GAK is a direct coregulator of AR. Recent studies have shown that the inhibition of the GAK kinase domain can inhibit the growth of PC cells [189,190].

## 5. The Role of PKs in Epigenetic Changes and Progression of PC 

Epigenetic disorders have been identified as a major factor in escaping cell death during cancer treatment and radiotherapy [191]. Epigenetic changes involve inherited and reversible changes in gene expression and mRNA translation without any modification of DNA sequences, which is considered as a link between phenotype and genotype [192]. In the epigenetic process, chemical groups have been added (writers) or removed (erasers) and recognized (readers) to alter gene expression after cell division and determine cellular fate. Epigenetic markers include DNA methylation, histone modification, chromatin remodeling, and noncoding RNA (ncRNA), especially for microRNAs (miRNAs) [193].

Epigenetic disorders have been reported to play a key role in the onset and progression of PC [194]. As many of the signaling pathways in advanced PC, including those involved in cell–to–cell adhesion, epithelial–mesenchymal transition, and the maintenance and regulation of stem cells, are epigenetically impaired, PC is considered as a cancer of the epigenome [195,196,197]. In fact, several enzymes, such as kinases contribute to these epigenetic abnormalities [198]. While the mechanism of PKs as cytoplasmic signaling transducers has been extensively studied, their roles as chromatin regulators are not as well-studied. The first evidence of a signaling kinase involvement in the direct regulation of chromatin in yeast found that key signaling kinase Hog1 was physically associated with promoter regions due to osmotic stress conditions. Since then, more evidence has demonstrated the role of kinases as epigenetic regulators that can modify transcriptional regulatory factors, histones, as well as histone modifiers in the nucleus. For example, the PKC family in the nucleus directly phosphorylates histones and transcription factors or forms complexes that associate with chromatin [199]. Akt, CDKs, PLK1, PKA, ataxia telangiectasia and Rad3-related kinase (ATR), and DNA-PK are the established kinases responsible for the phosphorylation of various epigenetic regulators. Epigenetic regulators undergo extensive post-translational modifications, in particular, phosphorylation. The deregulation of PKs can be frequently observed through neoplastic transformation and tumor progression. Therefore, kinases are required to be regulated via different genetic and epigenetic processes [194].

### 5.1. DNA Methylation and Histone Modification 

DNA methylation occurring by the DNA methyltransferase (DNMT) family [200] has been regarded as the most important epigenetic alteration [201], which plays a critical role in some biological phenomena, such as X chromosomal inactivation, differentiation, and genome imprinting during development. This phenomenon occurs mainly in cytosine residues in the C-phosphodiester-G (CpG) islands and suppresses gene expression [191]. Aberrant de novo methylation of CpG islands is a typical sign of human cancers and can be detected in the early stages of carcinogenesis [202]. 

Histone modification is another epigenetic change where the N-terminal tails of histones, in which lysine and arginine residues are located, target several post-translational modifications, including acetylation, phosphorylation, and methylation. Histone acetylation and deacetylation occur by histone acetyltransferase (HATs) and histone deacetylases (HDACs), respectively [194]. Depending on the modification position, the target gene is activated or suppressed [203]. 

Studies have shown that several epigenetic modifiers in cancer cells, including DNA methyltransferases, histone acetyltransferases, deacetylases, histone methyltransferases, and histone demethylases, are abnormally hyperphosphorylated or hypophosphorylated. Phosphorylation modification may directly suppress or activate these enzymes, indirectly regulate the interaction between modifiers with RNAs or proteins, or tighten or loosen the chromatin structure [194]. Among different epigenetic alterations, changes in the methylation of DNA are best identified and characterized in PC [16]. According to reliable evidence, the hypomethylation and hypermethylation of DNA occur in PC, leading to alterations in the methylation pattern in the tissue, and there is also a significant relationship between hypomethylation and hypermethylation with the progression of benign prostatic hyperplasia to metastatic tumors [192]. uPA causes tumor invasion and metastasis in some malignancies, such as PC. In highly invasive PC3 cells, the uPA promoter is hypomethylated [204]. The Ras family plays an important role as tumor suppressor proteins by activating the apoptosis process. This gene is commonly silenced through a methylated promotor in PC and several other cancers. Recently, the hypermethylation of Ras families has been observed in more than 70% of primary PCs) [205]. PTEN is a tumor suppressor gene located on chromosome 10. According to studies, the lack of PTEN activity has a profound effect on several Tyr kinases, such as PTK6 and Akt, that promotes PC progression [206,207]. Some evidence suggests that the epigenetic pathway is responsible for PTEN regulation and PTEN silencing in PC, which occurs through hypermethylation. PTEN can regain its activity by treatment with a DNA demethylating agent, such as azacitidine [198,208]. Furthermore, alteration in the methylation pattern of DNA is believed to be a significant source of tumor heterogeneity in metastatic PC and can lead to the development of therapeutic resistance [209]. 

Histone modifications, including acetylation, methylation, and phosphorylation, are other epigenetic alterations [210] that play an important role in the onset and progression of PC. The role of histone modifications has been identified in PC. For example, the main histone methyltransferase, responsible for H3K27 (the lysine residue at N-terminal position 27 of histone 3) trimethylation and the aberrant silencing of multiple tumor suppressor genes, namely the enhancer of zeste homolog 2 (EZH2), has been shown to be overexpressed in PC cells and hyperphosphorylated by several kinases, such as Akt, and thus promotes the expression of several critical oncogenes and induces PC metastasis [16,211]. 

Histone phosphorylation that depends on amino acids in histone is a dynamic process. Histone phosphorylation occurs by altering many cellular processes, including the cell cycle, repair of DNA damage, and cell apoptosis, so impaired regulation often leads to tumor formation. Hence, the kinases that regulate the phosphorylation of histones are always overexpressed in cancers. For example, high levels of PRK1, which mediates the phosphorylation of histone H3 (at Thr 11) [212], are associated with the advanced stages of PC [213]. In another study, Mahajan et al. reported that activated cdc42-associated Tyr kinase (ACK1) phosphorylates histone H4 at Tyr88 upstream of the AR transcription start site, leading to a WDR5/MLL2 complex-mediated increase in AR transcription. AR plays a major role in the onset and progression of PC. Therefore, the interaction between AR and ACK1 drives the positive feedback epigenetic circuitry that is ultimately conducive to promoting AR transcription. The inhibition of ACK1 reverses the phosphorylated Tyr88 at histone 4 (pY88-H4) marks and reduces AR and AR-V7 splice variant levels to mitigate castration-resistant prostate tumor growth [214,215].

### 5.2. MicroRNAs (miRNAs), as Epigenetic Modulators

There are several studies that clearly demonstrate the ability of miRNA in the epigenetic and post-translational regulation of gene expression. In PC progression, miRNAs play crucial roles through the regulation of kinase expression. In this review, some studies on this issue will be highlighted. It has been reported that the miR-135-a level was significantly reduced in metastatic PC tumors, indicating a correlation between tumor progression and a higher Gleason score. In fact, miR-135-a suppressed PC cell proliferation via the targeting of several oncogenic pathways, such as EGFR [216]. Another tumor-suppressive miRNA, miR-34c, plays a key role in PC through the targeting of the MET proto-oncogene. The MET proto-oncogene is a Tyr kinase family receptor that plays an important role in the invasion and migration of tumor cells. The upregulation of MET has been reported in metastatic tumors [217,218]. 

**Figure 3:**
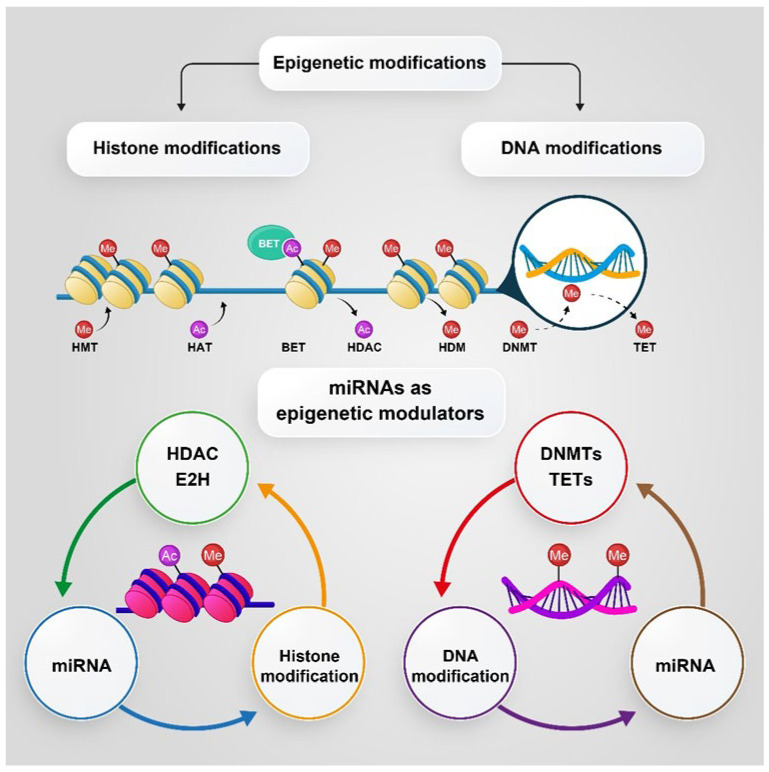
Epigenetic marks including DNA methylation, histone modification, and the relationship between miRNAs and epigenetics.

Other studies show that miR-139 and miR-302a downregulate Akt in PC. Cell cycle arrest through the upregulation of the CDK inhibitor p21 and downregulation of Akt and cyclin D1 has been attributed to the overexpression of mir-139 [219]. miRNA-302a also binds directly to the 3′UTR mRNA of the Akt gene, leading to the induction of cell cycle arrest in the G1/S phase [220]. Figure 3 shows the epigenetic regulation of DNA and histone modifications that are discussed above. 

## 6. PC Treatment and Management

As stated earlier, ADT and AR-targeted therapy are two gold-standard options for PC treatment [116]. Apart from AR blockade, immunotherapy, poly-ADP ribose polymerase inhibitors (PARPIs), and targeted therapies for prostate-specific membrane antigen (PSMA) are other options that have been developed for targeted therapies for PC, especially for the most aggressive, castration-resistant types. Nevertheless, after a while with these treatments, the tumor eventually develops resistance [221]. 

According to several studies, some pathways related to PKs, activated in the advanced stages of PC, are responsible for cases of resistance, and targeting these pathways may lead to overcoming the resistance to targeted AR treatment. For instance, mutations in the PTEN/PI3K/Akt signaling pathway are one of events responsible for resistance to PARPIs and PC progression [221]. Recent discoveries indicate that the crosstalk between this pathway and multiple signaling cascades can further promote PC progression and influence the sensitivity of PC cells to PI3K/Akt/mTOR-targeted approaches explored in the clinic, as well as standard treatments [112]. 

Although there has been a lot of progress in kinase drug discovery, many challenges remain in this field. As a challenge, tumors targeted by kinase inhibitors usually show resistance over time, leading to disease progression [33]. The search for targeted therapies of mCRPC has focused on developing new effective systemic treatments and identifying mechanisms of drug resistance [17]. Mounting evidence supports epigenetic events as potential mechanisms for PC transdifferentiation to an AR-indifferent state. Extensive studies have shown that DNA methylation plays a significant role in mediating these mechanisms in PC, among other cancers [12]. These are the key reasons why PK and epigenetic modulators have emerged as two combination therapy options to overcome acquired resistance to traditional therapies. However, understanding the mechanisms of synergy and resistance remains a crucial challenge.

### 6.1. PK Inhibitors in PC

PK inhibitor-based therapies exhibited a shift from conventional chemotherapy to targeted cancer therapy by overcoming a leading drawback of traditional cancer therapies. They effectively distinguish between normal cells and cancer cells [33]. Different PK inhibitors have been studied in various types of studies, including in vitro, in vivo, and clinical trials, in monotherapy or in combination with cytotoxic chemotherapy or radiation therapy, though with mixed results for mCRPC treatment [34]. About 20–25% of mCRPC subtypes that show somatic or germline alterations in DNA repair genes involved in homologous recombination are usually associated with more invasive disease. In the treatment of these subtypes, PARPI have shown significant effects. However, some epigenetic alterations or genetic mutations prevent PARP from binding to its inhibitors and consequently drug resistance. In PC, mutations in the PTEN/PI3K/Akt signaling pathway are one of the frequent events responsible for resistance to PARP inhibitors and disease progression [221]. As a result, several targeted PC therapies mainly affecting AR and the PI3K/Akt/mTOR pathway are in various stages of development [18]. Based on recent investigations, PTEN, PI3K, and PKB (Akt) inhibitors have offered promising results for mCRPC treatment with acquired resistance to PARP inhibitors, both in monotherapy and combined therapy with PARP inhibitors [221]. In a review article, Pungsrinont et al. summarized and discussed several inhibitors of the PI3K/Akt/mTOR pathway tested as monotherapy or in combination with other agents in preclinical and clinical trials for PC treatment [116]. Additionally, Shorning et al. presented new mechanical insights into the fundamental interaction between the PI3K/Akt/mTOR pathway and several oncogenic cascades (particularly the AR, MAPK, and WNT signaling cascades), which could facilitate PC growth and drug resistance [112]. Accordingly, Yadav et al. carried out a systematic study on the combined effect of therapies targeting the AR-signaling and the PI3K/AKT/mTOR pathways upon various PC cell lines. Their observation demonstrated that a combination of MDV3100 (AR-inhibitor) and BKM120 (PI3K-inhibitor) is highly synergistic. Furthermore, combining BKM120 with TKI258 (pan RTK inhibitor) has better synergy than BKM120+RAD001 (mTOR inhibitor) or RAD001+TKI258 in all of the lines, irrespective of androgen sensitivity. Finally, the PI3K inhibitor also displayed synergy when combined with the chemotherapy drug cabazitaxel [18]. 

Other potential targets, including ATR, CHK1, WEE1, AURK, and PlK1 have been successfully examined in preclinical studies for PC treatment [220]. Several preclinical studies showed that the association of PARP inhibitors with ATR inhibitors could resensitize PARP-resistant cells [222,223,224]. Neeb et al. characterized ATM-deficient lethal PC and studied ATR inhibition, PARP inhibition, and combined PARP and ATR inhibition as therapeutic strategies for this subset. They found variable sensitivity of this subtype to PARP inhibition, sensitivity to ATR inhibition, and the most sensitivity to combined inhibition, which now merits clinical evaluation [225]. 

Currently, some pK inhibitors investigated in mCRPC clinical trials include dasatinib [226,227], trametinib [228], masitinib [229], sunitinib [230,231], bevacizumab [232], cediranib [233], cabozantinib [234], erlotinib [235], and ipatasertib [236,237,238] (Table 1). Of all the agents presented, ipatasertib has shown excellent preliminary therapeutic results and a favorable safety profile (in both early-phase and late-phase testing) in patients who have lost PTEN, and it may be a good combination partner with multiple anticancer agents [236,237,238]. The upregulation of the RAS pathway following ipatasertib suggests that the coadministration of ipatasertib with the inhibitors of the RAS/MEK pathway may be more effective [236]. For all other agents, further definitive testing to clearly evaluate their clinical potential has been recommended.

### 6.2. Epigenetic Targeting as a Therapeutic Strategy for Advanced PC 

As previously stated, pieces of evidence from several studies suggest a cross-link between kinase pathways and epigenetic reprogramming during the progression of PC. In our opinion, this evidence opens an opportunity to develop new strategies in PC treatment and management, particularly for patients with developed CRPC and AR-indifferent forms of the disease. 

In a review article published by Angus et al. [33], they highlighted the epigenetic changes underlying resistant phenotypes and discussed phenotypic switching as an adaptive response to kinase inhibition. They mentioned that developed strategies are needed to block the dynamic changes in the chromatin landscape in response to kinase inhibitors, leading to adaptive resistance and durable responses. Thus, they suggested that the small-molecule inhibitors of these epigenetic regulators have the potential to attenuate the transcriptional rewiring that leads to drug resistance. Finally, they proposed potential therapeutic approaches by the combination of targeting key oncogenic kinases with drugs targeting the components of the transcriptional machinery and histone-modifying enzymes. 

**Figure 4:**
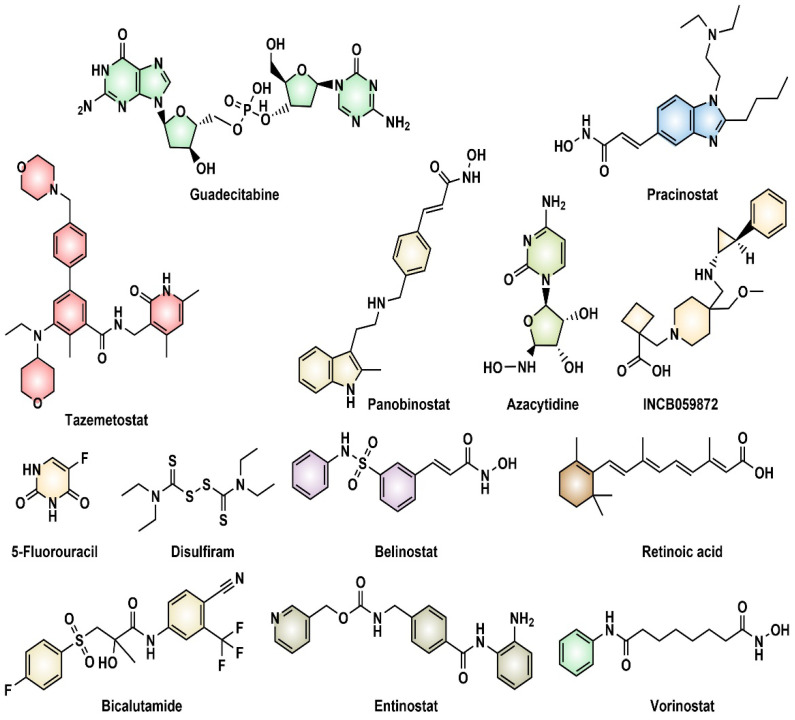
Some epigenetic inhibitors that have shown activity in prostate cancer.

Currently, several epigenetic inhibitors are under preclinical and clinical trials for the management and treatment of PC. They include histone methyltransferase inhibitors, DNMT inhibitors, HDAC inhibitors, and many other numerous epigenetic therapies, which are currently under preclinical and clinical investigations for the management and treatment of PC. Some epigenetic drugs that are reported from active and completed clinical trials and used to treat PC include: tazemetostat as an EZH2 inhibitor; guadecitabine, disulfiram, and azacitidine as DNMT inhibitors; belinostat, entinostat, vorinostat, and panobinostat; HDAC inhibitor SB939, 5-fluorouracil, and bicalutamide as HDAC inhibitors; and nivolumab, INCB059872, all-trans retinoic acid, and azacytidine as lysine-specific histone demethylase 1 inhibitors [16]. These epigenetic inhibitors are depicted in Figure 4.

## 7. Conclusions

Due to acquired resistance to conventional treatments for PC, including radiotherapy, prostatectomy, and androgen deprivation, recently, novel treatments, including targeting several signaling pathways and epigenetic modifiers, are in development. As mentioned above, different PKs play a significant role in several pathways related to PC progression; hence, PK inhibitors may be suggested as potential therapeutic agents in PC. The promising clinical results of phases I, II, and III on ipatasertib show that this Akt inhibitor can be a good combination partner with several anti-cancer agents for mCRPC treatment. Given that many of the signaling pathways are epigenetically dysregulated in PC, epigenetic targeting may represent an alternative therapeutic strategy to treat advanced PC with genetic modulator inhibitors. Additionally, various studies show that there is an interplay between PKs and epigenetic changes in PC; thus, it seems that simultaneously targeting these pathways may be a suitable treatment option for advanced PC.

## Figures and Tables

**Table 1:** Summary of several active and completed clinical trials evaluating the efficacy of some kinase inhibitors in monotherapy and in combination with other treatments in mCRPC.

Compound	Target	Result	Type of Study	Reference
Dasatinib	SRC Tyr kinase family	✓ Poorly tolerated and limited activity in advanced mCRPC patients who were treated previously with chemotherapy ✓ Considerable toxicity that limits its broad application ✓ Observation of a case with a prolonged objective response and clinical benefit warrants molecular profiling to select the appropriate patient population	Phase II trial	[226]
		✓ Addition of dasatinib to docetaxel did not improve median overall survival.	Phase III trial	[227]
Trametinib	MAPK	✓ Observation of a case with biochemical and clinical response in a patient experiencing failure of several previous treatments for mCRPC	Phase II trial	[228]
Masitinib	FAK	✓ Favorable and compatible safety profile of masitinib with a long-term regimen at 12 mg/kg/day ✓ Tumor control rate in imatinib-resistant patients was encouraging ✓ The maximum tolerated dose was not reached, and the acceptable dose was identified at 12 mg/kg/day.	Phase I trial	[229]
Sunitinib	RTK	✓ Common adverse effects included transaminase elevation, nausea, fatigue, diarrhea, and myelosuppression. ✓ Only 1 of 17 patients showed a 50% decline in PSA and radiographic measurements of disease were discordant, indicating that alternate end points are important in future trials.	Phase II trial	[230]
		✓ Addition of sunitinib to prednisone did not improve median overall survival. ✓ Common adverse effects included fatigue, asthenia, and hand–foot syndrome.	Phase II trial	[231]
Bevacizumab	VEGFR Tyr kinase	✓ Despite an improvement in median progression-free survival and objective response in men with mCRPC, the addition of bevacizumab to docetaxel and prednisone did not improve overall survival and was associated with greater toxicity.	Phase III trial	[232]
Cediranib	RTK	✓ Well tolerated with anti-tumour effect in mCRPC patients who had progressive disease after docetaxel-based therapy ✓ Common adverse effects included hypertension, weight loss, anorexia, and fatigue; the addition of prednisone reduced the toxicity.	Phase II trial	[233]
Cabozantinib	RTK	✓ Cabozantinib did not significantly improve overall survival	Phase III trial	[234]
Erlotinib	VEGFR Tyr kinase	✓ Moderate toxicity ✓ No patient had a decrease in PSA and 14% had stabilization, less than the ≥20% expected. ✓ Clinical benefit was achieved in 40% of patients.	Phase II trial	[235]
Ipatasertib	Akt	✓ Ipatasertib monotherapy demonstrated a favorable safety profile and preliminary antitumor activity (30%)	Phase I trial	[236]
		✓ In the *PTEN*-loss group, patients demonstrated improved radiographic progression-free survival compared to those without *PTEN* loss.	Phase II trial	[237]
		✓ Ipatasertib plus abiraterone/prednisone demonstrated that radiographic progression-free survival was improved in the *PTEN*-loss population. ✓ Overall survival and other secondary endpoint data are awaited.	Phase III trial	[238]

## Data Availability

No new data were created or analyzed in this study. Data sharing does not apply to this article.
